# Supplementary material for: Sonic Hedgehog Pathway Modulation Normalizes Expression of Olig2 in Rostrally Patterned NPCs With Trisomy 21
Source: Front Cell Neurosci. 2022 Jan 4;15:794675. doi: 10.3389/fncel.2021.794675 (PMC8763807; doi:10.3389/fncel.2021.794675)
Supplement: Supplementary file 1 [file Data_Sheet_1.pdf]

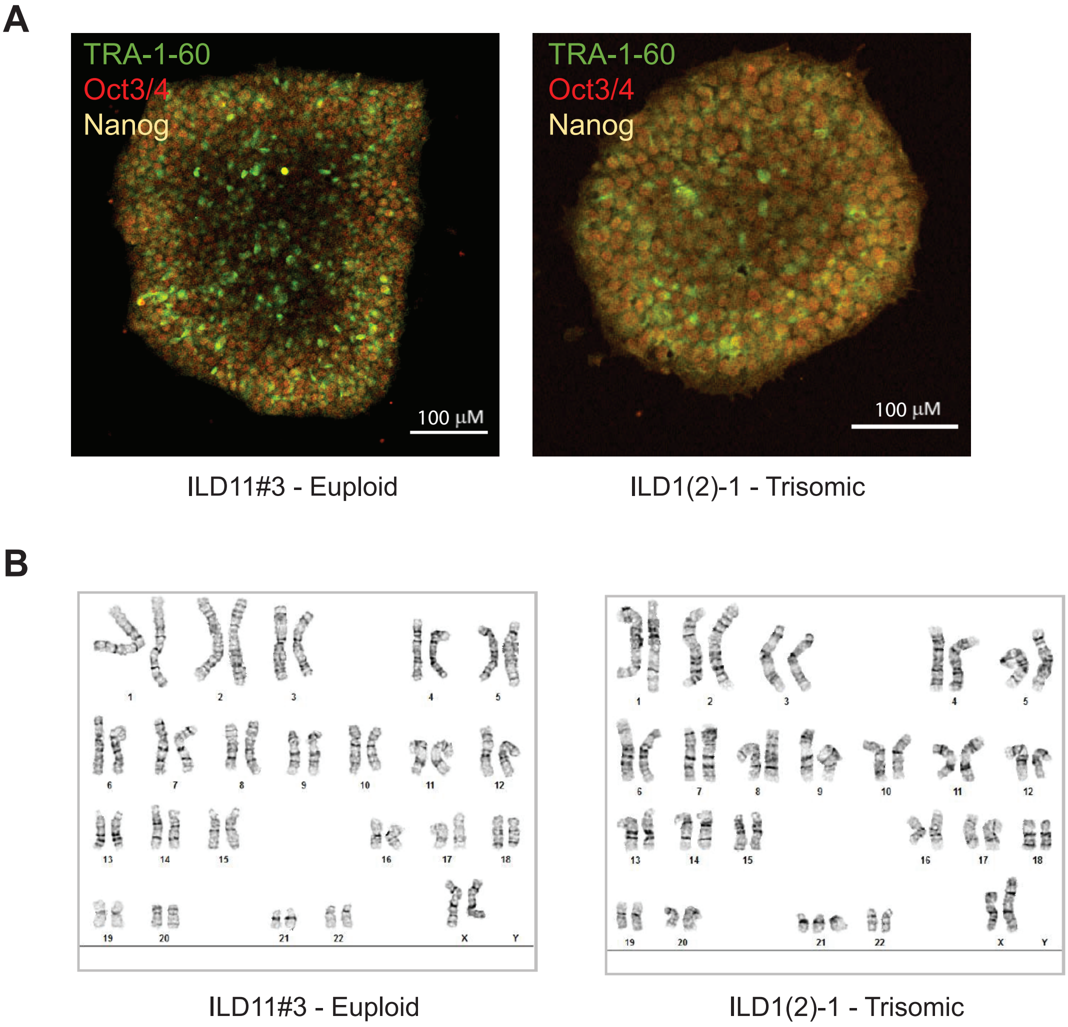

Supplemental Figure 1 - Validation of ILD isogenic iPSC lines. A) Pluripotency staining confirming expression of TRA-1-60, Oct3/4 and Nanog. **B)** Both lines were derived from the same renal epithelial cell line obtained from an individual with Down syndrome. Upon karyotyping, a euploid clone was identified (left). Short tandem repeat analysis confirmed identical origin for both lines.

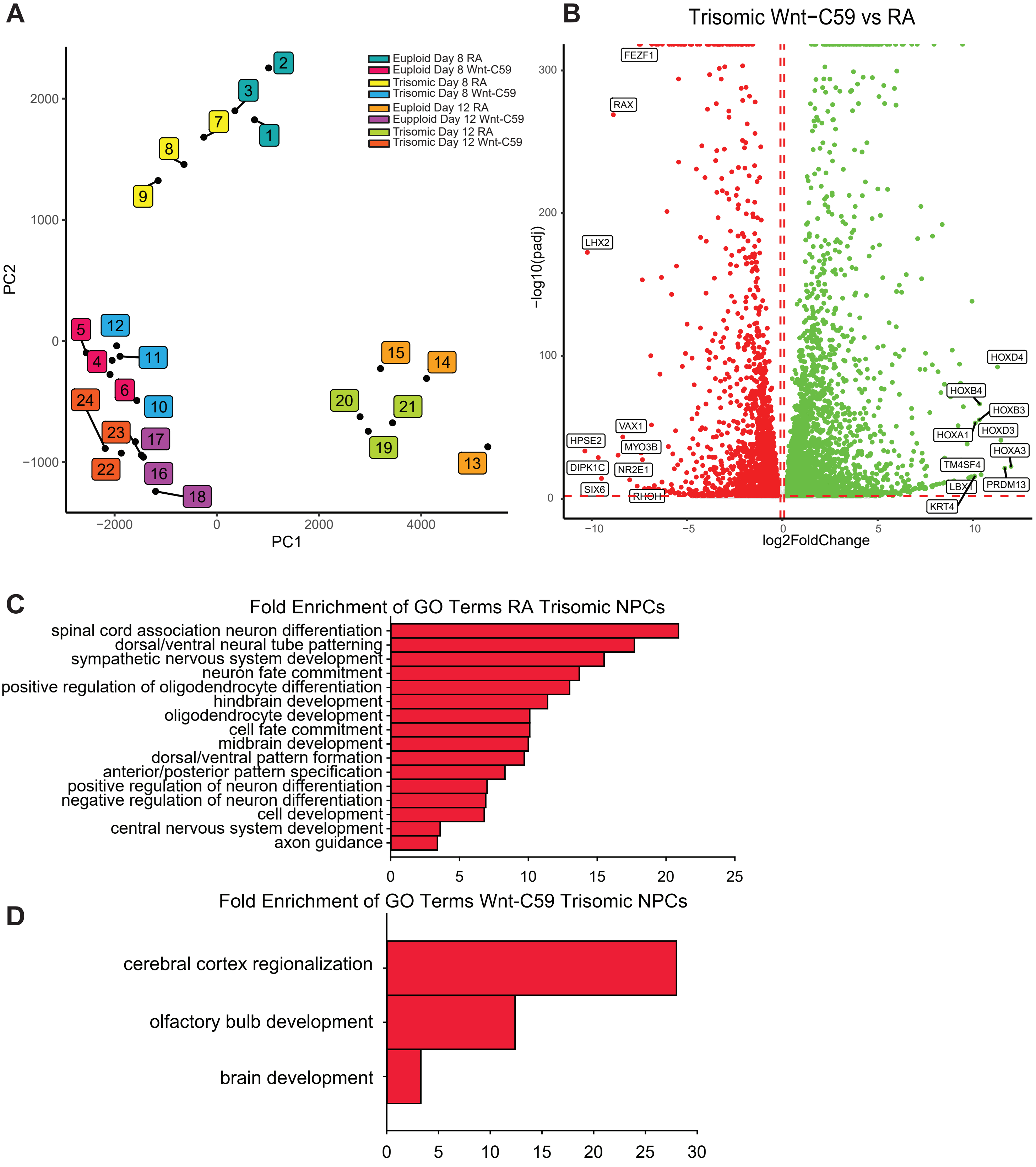

Supplemental Figure 2 – RNA-seq clustering and trisomic NPC regional patterning. A) PCA plot of all 24 RNA samples collected for RNA-seq analysis. PC1 explains 67.4% of the observed variation while PC2 explains 14.2%. The triplicates of each condition cluster together and we note that condition (Wnt-C59 vs RA) and day (8 vs. 12 in RA) drive the clustering more than genotype. **B)**10,115 genes are differentially expressed in trisomic NPCs differentiated with either RA or Wnt-C59. 5,169 are upregulated in the Wnt-C59 condition and 4,946 in the RA condition. **C)** GO analysis identifies biological processes significantly enriched in Wnt-C59 trisomic NPCs relating to the rostral CNS. **D)** GO analysis identifies biological processes relating the caudal CNS significantly enriched in trisomic NPCs treated with RA.

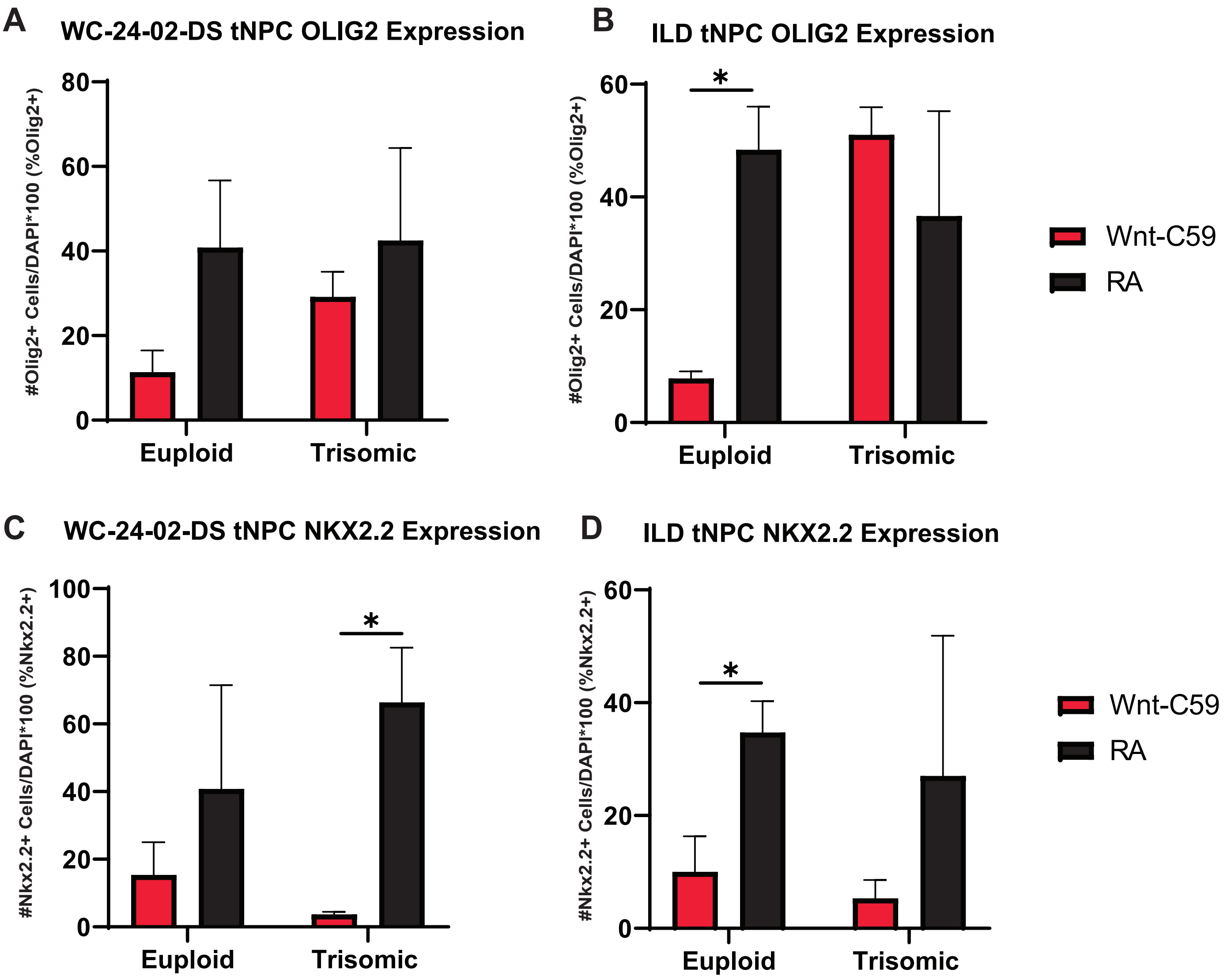

Supplemental Figure 3 – Comparison of OLIG2 and NKX2.2 expression between patterning conditions. A) There is no difference between the RA and Wnt-C59 conditions in percentage of cells expressing OLIG2 in either genotype in the WC-24-02-DS line. **B)** In the ILD isogenic line, the Wnt-C59 euploid condition has significantly fewer OLIG2+ cells than the RA condition. **C) I**n the WC-24-02-DS line, the Wnt-C59 condition generally leads to lower NKX2.2 expression than the RA condition, though it is only significant in the trisomic genotype. **D)** In the ILD line, the Wnt-C59 condition also shows less NKX2.2 expression in both genotypes though neither are significant. n=3 independent differentiation experiments, * p-value < 0.05 with specific values in text.

A

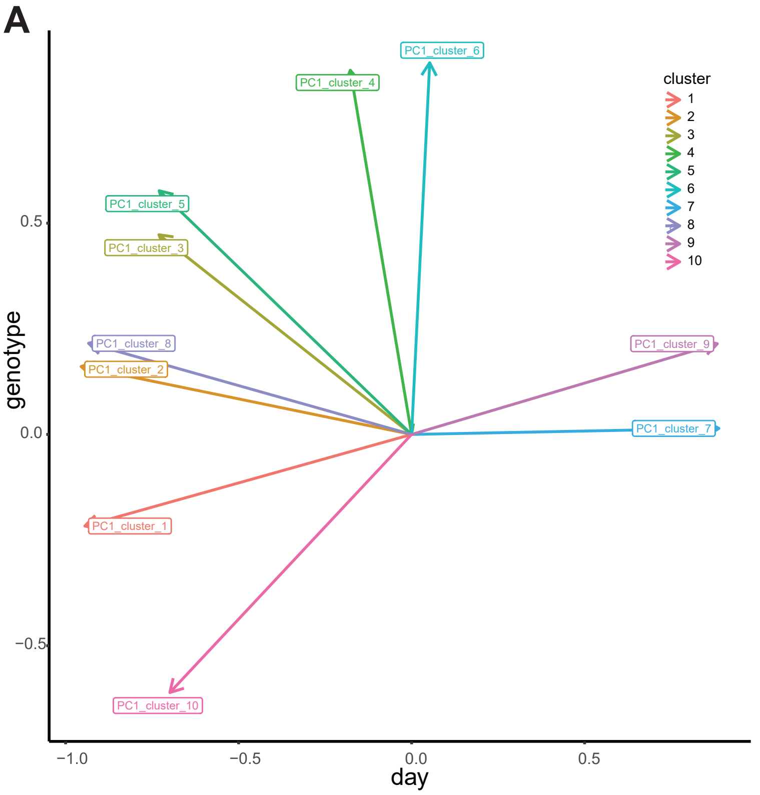

B

Cluster	%Variation in PC1	Correlation to Day
1	95.248	-0.94445
2	93.557	0.955467
3	99.343	-0.73042
4	95.448	-0.17723
5	78.99	0.729715
6	91.997	0.052246
7	95.035	-0.8882
8	93.708	0.933868
9	88.52	0.883743
10	89.007	-0.69881

C

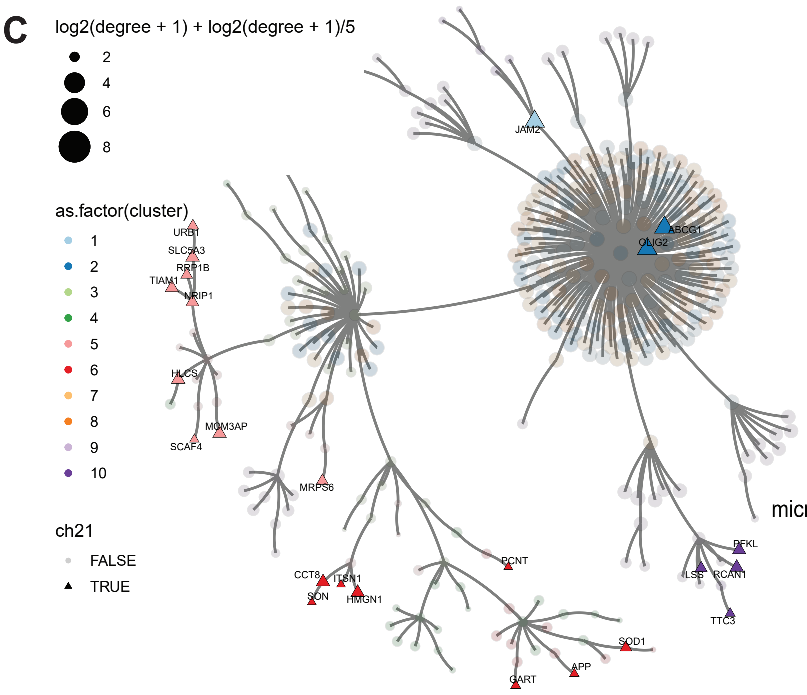

D

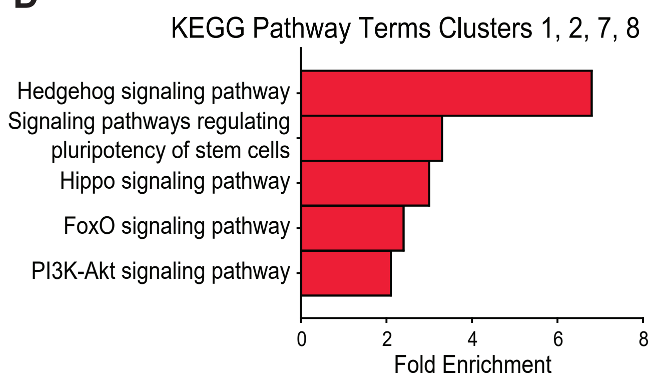

E

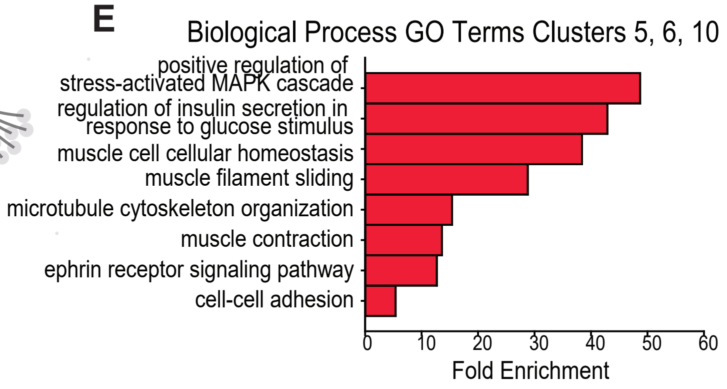

F

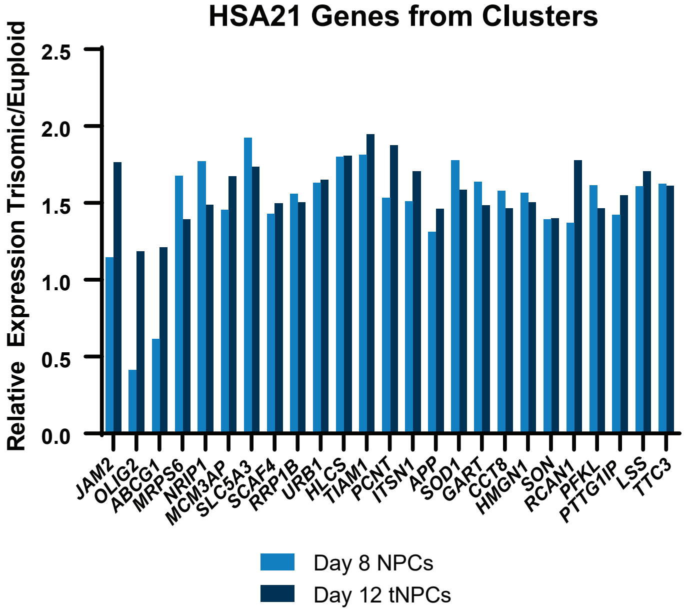

Supplemental Figure 4 – Additional RNA-seq and hierarchal clustering analysis. RNA sequencing and analysis was performed on the WC isogenic lines patterned with Wnt-C59 at both day 8 (NPCs) and day 12 (tNPCs). **A)** Arrows showing the correlation of principal component 1 (PC1) of each gene cluster to either day or genotype. **B)** Quantifica-tion of the percent variance explained by PC1 and its correlation to differentiation day. **C)** Location of HSA21 genes on the pairwise correlation plot. The majority of genes are peripheral way from the central node. **D)** GO analysis of the genes in clusters 1, 2, 7, and 8 identified significantly enriched KEGG pathways including hedgehog signaling as the most enriched. **E)** Gene ontology analysis of the genes in clusters 5, 6, and 10 where the genes are consistently overexpressed in the trisomic cells show multiple terms related to the cytoskeleton and extracellular matrix. **F)** The 25 genes identified in the ten clusters that are located on HSA21 are all significantly differentially expressed at approximately 1.5x euploid as expected due to gene dosage though the expression varies between genes and day.

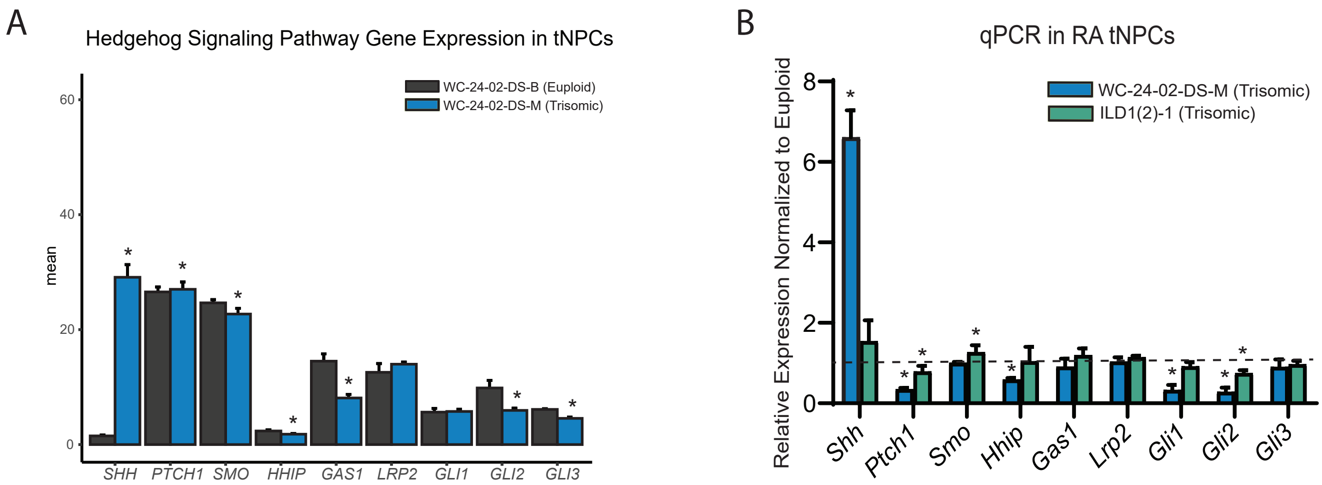

Supplemental Figure 5 – SHH pathway gene dysregulation in RA patterned tNPCs. A) Genes in the SHH signaling pathway in our RNA-seq dataset shows that there are significant differences in expression in many of them in the trisomic tNPCs differentiated with RA. **B)** qRT-PCR validation of the SHH pathway genes from the RNA-seq dataset show dysregulation in both trisomic lines (WCts and ILDts) of differentiated tNPCs compared to their euploid controls (WCeu and ILDeu). n=3-5 independently cultured wells. * p-value < 0.05 with specific values in text.

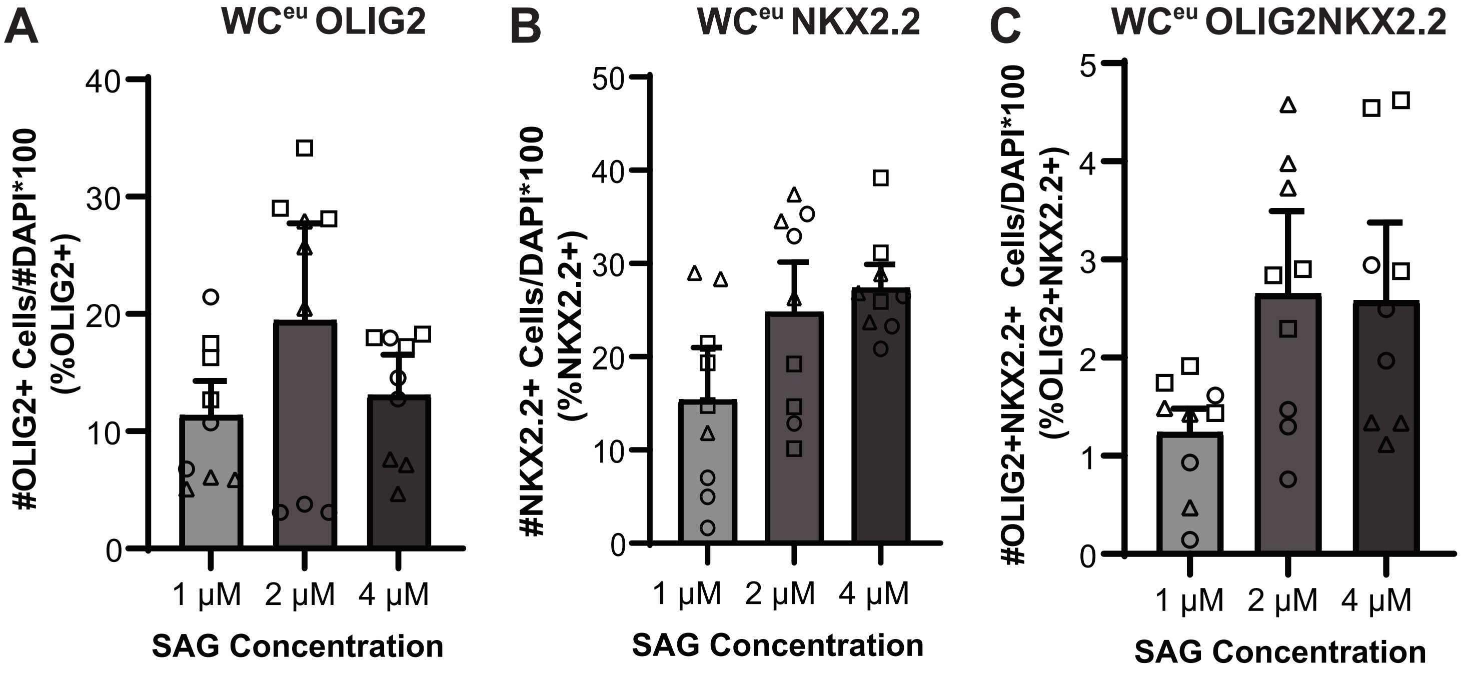

Supplemental Figure 6 – **Increasing SAG concentration does not change OLIG2 and NKX2.2 expression in euploid tNPCs.** In opposition to the trisomic cultures, increasing the concentra-tion of SAG in the euploid WC line cultures from 1 μM to 2 μM and 4 μM does not signifi-cantly change the percentage of cells expressing **A)** OLIG2, **B)** NKX2.2, or **C)** co-express-ing both proteins from their baseline expression in the 1 μM condition. n=3,3 independent differentiation experiments for all IHC measurements with values for individual wells over-laid. Note that the 1μM data is the same as Figure 3 and was performed independently from the 2 μM and 4 μM data. Data is shown as mean ± SEM.

Supplemental Table 1 - Computed cell counts were compared against the hand-counts for the OLIG2 and NKX2.2 stains. The DAPI stain marks too many cells and cannot be collapsed into a 2D image and will be further tested in the 3D version of the cell counting algorithm. The average error for the 2D version of the algorithm from this initial testing is 4.53%.

Hand-Count	2D App Cell Count	Percent Error (%)
121	121	0
187	196	4.8
153	152	0.65
382	356	6.8
332	335	0.9
265	263	0.7
39	44	14
381	402	5.5
177	168	5.1
316	297	6.0
335	317	5.4
303	317	4.6
**Average Percent Error**		4.53%

Supplemental Table 2 - Computed cell counts were compared against the hand-counts for the DAPI, SATB2, and CTIP2 stains. The average percent error for the 3D version of the algorithm was 4.25%.

Hand-count	MATLAB 3D Cell-Counting Algorithm	Percent Error (%)
819	783	4.40
214	219	2.34
214	214	0.00
663	710	7.08
97	109	12.37
295	293	0.68
684	755	10.38
227	239	5.29
115	118	2.61
**Average Percent Error**		4.25

Supplemental Table 3 - Catalog numbers for probes for qRT-PCR experiments. All probes are labeled with FAM dye except for UBC which is labeled with VIC.

**Gene Target**	**ThermoFisher Scientific Catalog Number**
Olig2	4453320
Nkx2.2	4453320
Shh	4453320
Ptch1	4453320
Smo	4453320
Hhip	4453320
Gas1	4448892
Lrp2	4453320
Gli1	4453320
Gli2	4453320
Gli3	4453320
Axin2	4453320
GAPDH	4331182
UBC	4453320

Supplemental Table 4 - Olig2 and Nkx2.2 protein co-expression as measured by IHC in each experimental condition and iPSC line.

Condition	Cell Line	%Olig2/Nkx2.2 Co-expression (± SEM)
Retinoic Acid	WC^{eu}	8.8% (± 5.5%)
	WC^{ts}	15.3% (± 4.7%)
	ILD^{eu}	9.4% (± 1.8%)
	ILD^{ts}	10.4% (± 6.7%)
Wnt-C59 1 µM SAG	WC^{eu}	1.2% (± 0.2%)
	WC^{ts}	0.9% (± 0.2%)
	ILD^{eu}	0.6% (± 0.2%)
	ILD^{ts}	1.2% (± 0.9%)
Wnt-C59 2 µM SAG	WC^{ts}	2.0% (± 0.5%)
	ILD^{ts}	0.8% (± 0.3%)
Wnt-C59 4 µM SAG	WC^{ts}	1.0% (± 0.2%)
	ILD^{ts}	0.6% (± 0.3%)

Supplemental Table 5 – The top 1000 DEX genes identified from likelihood ratio test as significantly different during the euploid and trisomic NPC transition response to SAG. The genes and their assigned cluster from hierarchal clustering are listed. These genes were used in the analyses shown in Figures 5 and 6.

Cluster Number	Genes in Cluster
1	*SHH, RUNX1T1, TGFB1I1, ETS1, HSPB8, NRK, CCN2, ZNF536, GADD45B, MYORG, FOSL1, CLDN6, BFSP1, SLIT2, SGK1, LHX8, TGFB2, RFTN1, SLC32A1, EDN1, S100A10, GLIPR1, KIFC3, L1CAM, POU3F4, HBEGF, MYOF, CAV1, TAGLN2, CRIM1, NKX2-4, DDAH1, SCARF2, CDKN2B, POU3F3, CSRNP1, BMP1, NOX4, PDLIM1, ADGRV1, PDE4D, KLF6, ABAT, TMTC2, SPON1, SHD, JAM2, H2BC21, SYNE1, TNRC18, SCUBE3, HIVEP3, SPARC, LHX6, BAHCC1, RUSC2, CUX2, GPC3, KIF5A, COL18A1, POU2F2, PCSK1N, TTC7B, AKAP12, TEX2, SLC27A4, ATCAY, TET3, PEG10, FAM171A1, NOL4, PSD, BCL2, HSPB1, DCT, HSPA5, EZR, MVD, SIX6, SETBP1, B4GALNT1, GPC4, TANC2, KIF3C, GPR50, ADAM23, MPPED2, MYO16, PPIB, SCN3B, CCDC85C, SYT1, TMEM151B, AP1S2, PAG1, ANKRD50, CYYR1, WNK2, IGDCC3, NPTXR, SLC39A6, AFF2, SOX6, SQLE, CDHR1, CRMP1, RTN2, SYNJ1, CRHBP, CANX, ECE1, NEDD4, TCF7L2, TSPAN17, FAT3, LOX*
2	*NEUROG3, STMN2, SVOP, INSM1, OLIG1, ADGRA1, SYT4, ST18, ELAVL4, DLL3, ELAVL3, SEMA3C, FGF5, SCRT2, FOXD1, CPLX2, OTP, ISL1, ACTL6B, PMEPA1, ASCL1, FOXC2, SYT5, EMP1, AMER2, ARX, FOXB1, OLIG2, SHB, SYP, HEY1, ADGRA2, SOHLH2, FGFR3, GADD45G, OLFML2A, GAP43, BHLHE40, C14orf132, DLX1, SRRM4, CRYL1, FAM181B, COL8A1, SYNPO, TCIM, THSD7A, FREM1, GSX1, ITGA11, CERCAM, FGFR2, ZCCHC12, FOXD2, XKR7, RASD1, GAD1, HELT, ABCG1, IL11, LBH, CDKN1C, NTN1, CPEB2, ISLR2, RFX4, PHACTR3, LIN7A, DLEU7, SYBU, SMPD3, PIM1, F2RL1, TLE6, TRIM67, UNC13A, CCND1, AUTS2, NEFM, CDK5R2, FBXL16, TLE2, ERBB4, CELF3, NFASC, HHIP, STRA6, DLX5, CHST15, DPYD, PLXND1, ACKR3, CRIP2, RAB11FIP4, LDLRAD4, PTPRN, LHFPL6, EPHB3, PRMT8, CACNG4, PLAGL1, GAD2, SMAD7, HES1, TP53INP2, DCC, SPARCL1, LZTS1, NCR3LG1, ADGRG6, P4HA2, KIF21B, DLX2, ADAMTS3, CNR1, RUNDC3A, CDC25B, MICAL2, ZMAT3, COL5A1, RNF150, RND3, COL1A1, KCNG1, BMP7, MGST1, PWWP2B, SLC18B1, MYT1, BAIAP3, SLCO5A1, AMOT, TGFB1, GSE1, NKX2-2, GASK1B, RNF182, PRKCA, NOG, RNF152, CELSR1, GSX2, SFTA3, STK39, ANKRD6, FGD3, CKB, RHOB, HCRT, GDI1, RHOBTB3, EEF1A2, FRMD6, DCX, SCN3A, SERPINE2, GNG2, KISS1R, DUSP1, STMN3, NDRG2, FZD9, SREBF1, CNTN2, ACSS2, PDZD4, SEMA6D, ITGA1, ADCYAP1R1, FOXA2, ABCA1, TRPS1, JUND, SFXN5, JUNB, MAP6, DHCR24, ASAP3, GALNT17, GSTO1, TUBB4A, CADM1, DENND4B, ITPR2, BAG2, NMNAT2, NRCAM, DLX6, PBX3, PAPPA, SNX30, HLA-B, TLE5, ZDHHC22, ITGA2, NHSL2, ABCA5, PDGFC, MBIP, CHD3, GNAO1, DPYSL4, NKX2-8, SOX3, HES6, CD83, ZFHX3, NEFL, IGFBP7, VAX1, SSBP3, LDLR, KCNJ8, AK3, LIX1L, CBL, PCSK9, KIT, ABCD1, EDNRB, SDK1, SEPTIN3, VWA5B1, SLC25A12, EPB41L3, FAM210B, SHISAL1, CADM3, PLA2G3, PGPEP1, RHBDL3, HSDL2, ROBO2, CDKN1A, OS9, CDK5R1, RTN1, WDR47, KCNN1, IGF1R, GDAP1L1, ANTXR1, EFHD2, SP9, PLEKHA7, TAOK3, MACIR, PAM, BACE2, SHISA7, MN1, DNAJA1, CHIC2, CHGB, TMEM132E, HID1, MMP2, MDGA1, NPAS3, DIRAS1, MOXD1, OLFM1, PRKX, TAGLN3, HDAC9, PDPN, TOX2, HMGCR, PRCP, FHL1, NKX2-1, TTYH2, FBXO7, WSB2, SREBF2, ID3, NSDHL, ZNF462, KIAA1549L, FADS2, PGRMC1, GPSM2, APH1B, BMPR2, FASN, SPTLC2, DHCR7, LITAF, RBPMS2, GPM6A, GREB1, RNF208, FGFBP3, RIMS4, FABP3, JAK1, TUBA1A, NEGR1, FMNL2, OLFM2, RTN4, MEST, AGO1, CXCL14, CD24, FDPS, ETNK2, RTL5, KIFAP3, ATRNL1, REEP1, MAGED1, MMRN1, MAP1B, CRB1, ATXN1, PSME1, MDM2, PTCH2, RRAGD, HMGCS1, SPAG9, GRIK3, RBP1, GLI1, KIF1B, ACAA2, ITGA6, VEGFB, FOXN4, PRNP, PREP, NHLH2, EPHB2, ELF4, ITGAV, SPART, ETFB, IRF2BP2, PSD3, TLE3, MEIS3, CHST11, TERF2IP, MANEAL, FDFT1, ITPRIP, NHSL1, CAND2, INPP5A, MARK1, NKAIN1, GDI2, FBLN1, ARID5B, EEPD1, STXBP1, HSPA4L, SCG3, RAB3A, PLPPR2, SCD, SLC44A5, FOXO1, SPIRE1, TIPARP, FST, VIM, TMEM135, ELOVL6, SEC23A, DCLK1, ZHX2, RDX, PNPLA3, EPHB1, ACVR2A, ACAT2, IL13RA1, DAZAP2, SLC25A20, TBCA,*

	SNAP91, STMN1, CCDC8, ULK2, ZEB2, EFCAB2, ACSL3, LRRC17, FADS1, DYNC1I2, TMEM98, LAMP2, BAZ1A, HIVEP2, TMEM97, ABHD3, EBF4, APC, SRGAP1, SKIL, PCYT2, ARL4D, ACSL1, C1QL1, MCC, MARCHF5, LPGAT1, IBTK, ITM2B, GPRIN1, DACT3, INSIG1, UHRF1BP1L, CHPT1, ATP1B3, MFSD2A, SNAP25, PTCH1, AK4, ELAVL2, BCAM
3	*LEFTY2, GPAT3, NKX6-2, CHRNA4, SCG2, NSG2, ONECUT3, VCAM1, SOCS2, FGF19, SLIT1, TCEAL7, SLC25A5, FGF13, PMAIP1, RAB33A, NNAT, IGFBPL1, NTRK2, NKX6-1, TCEAL9, GNB4, ATP11C, RADX, NPTX2, PREX1, CXCR4*
4	*HMX1, UNCX, TBX1, NHLH1, NEUROG1, ROBO3, FABP7, H1-0, BEX2, TCEAL8, PCDHGB4, BEX4, SLC15A4, TSPAN6, PCDHB5, PRPS1, KLHL13, POU6F2, ZDHHC9, BEX3, OCRL, SCRN1*
5	*ACTA1, TPM1, CNN2, TMEM47, PCSK5, MRPS6, SHROOM2, NRIP1, CLCN4, GPR143, MCM3AP, HTT, DMD, SLC5A3, TIAM1, SCAF4, GJA1, PLXDC2, RRP1B, URB1, SHISA2, TBL1X, HLCS, EFNA5*
6	*PTPRN2, GATD3B, ZNF558, FP565260.1, SLITRK4, PCNT, TRIM61, NLRP2, ITSN1, APP, SOD1, GART, RPS4X, CCT8, CHCHD2, HMGN1, SON*
7	*EMX2, RBM47, COL9A3, TMEM163, ETV4, ZIC5, VRTN, SHISA6, MEIS1, CA14, HPSE2, HS3ST3B1, RSPO1, FGF8, HS3ST3A1, ZIC2, CA4, SEZ6L, SLC7A11, IL1RAPL1, SIPA1L2, ZNF385B, SERINC5, TRANK1, IGFBP5, BST2, CYP2S1, CA2, ANKRD35, ZNF217, QPRT, WIPF3, GPR176, SHMT2, CD82, TP53I11, IGSF1, C6orf141, STOM, ERAP1, NUAK2, SORL1, KRT19, GLI2, PYGL, ZNF185, DHX32, ME3, SMOC1, COLGALT2, RHOU, FOXO3, GCNT1, PCDH18, SEMA3A, LCTL, ROR1, NAV1, ADD3, SLC6A9, UHRF1BP1, NCALD, LRP2, PRRT3, ERBB3, PCYT1B, TMEM169, IFITM2, COL4A5, ROR2, GALNT12, ENC1, CNTNAP2, CDH11, COL4A6, ZNF521, SPRY2, ADGRL1, EPHA4, MARS1, IRS4, VIPR2, COL2A1, JAG1, PROM1, STC2, PTPRZ1, SNTB1, KCNJ5, ITGB5, FZD5, SPRY4, CDH8, RASSF3, ADM2, L1TD1, NAB1, XPNPEP1, VAT1L, GPRASP1, SPRY1, ARAP3, PKDCC, ARMCX2, NEK9, DLK1, LAMA1, PCDHGC4, MFAP2, SOX21, CPXM1, TBC1D9, MYO5C, ETV5, GPT2, ATP2B1, AFF1, SLC29A1, C1orf226, ROBO1, NR6A1, FRY, TRAF5, IFITM3, DMKN, SCUBE1, TRIM45, GPR153, GPD2, TNKS1BP1, TNNT1, SEL1L3, GRAMD1B, COL26A1, FUT8, MECOM, BLACAT1, USP54, PTGIS, DMRTA1, CLU, PTN, FHDC1, CACHD1, PTPRD, OGA, ITGA5, TACC2, GLI3*
8	*EMX1, LAMP5, PAX6, PAMR1, PPP2R2B, EGF, CDH20, CLEC19A, ATP1A2, ANOS1, IGFBP3, KCNK12, WNT7B, ARID3C, UNC5D, VCL, TMEM88, DDIT4L, ADAMTS9, ALCAM, MYH2, NPY2R, GAS1, AJUBA, NRP1, SHISAL2B, UGT3A2, PAX8, TMEM74, ATF5, HS6ST2, TPM2, ID2, SEMA5A, SORCS1, LSR, ARRDC4, FBN3, PALLD, FLRT3, MYO3B, CD74, KIRREL1, SLC16A9, ARHGAP45, ALDH2, LLGL2, LPAR4, EFEMP1, GREB1L, ADCY6, NLGN1, TXNIP, DIPK1C, TRPM3, SAMD12, SFRP2, SERPINH1, NID1, POGLUT3, WNT5B, RAX, PTPRG, SEMA3F, KLHDC8A, CCDC177, SYT17, PLEKHG3, SLC2A1, MAPKAPK3, MYLK, TRIL, LIN28A, SP8, MAPK10, ZNF608, SALL2, WNT2B, GLDC, RCN1, SCUBE2, MARVELD1, FAM124A, AHDC1, PLEKHG4B, SALL4, DYNLL2, TBC1D30, PALM2AKAP2, TNS3, LAMB1, CDON, SLC7A8, HELLS, DIAPH1, FZD1, SPHK1, DNMT3B, CECR2, CYBA, ZBTB16, LMAN1, SLC6A8, ERRFI1, COL11A1, SULF1, MYO5B, LRIG1, CAD, LRBA, LFNG, SART3, TYRO3, B4GALNT3, LIX1, PTPN13, FEZF1, MCF2L, KDM2B, TBC1D1, FBXW4, HOMER1, DENND2B, TRIM71, PRSS23, ZBED3, ZNF544, LIMK2, OTX1, WASF3, PAPLN, USP44, CLCN2, HECTD1, NREP, MAP2K6, IL17RD, MYO10, ZNF93, ATXN7, HEPH, NECTIN1, SOX2, UCHL1, MAN2A1, CEMIP2, ALPL, FBN1*

9	*CRABP1, EOMES, IFITM1, FEZF2, CHAC1, BOC, PTGR1, SERPINF1, DDIT4, ULBP1, ZIC4, VEGFA, DSP, PLPP3, INHBE, TSPYL5, BCAT1, PCK2, PSAT1, ARHGEF4, MTHFD2, UBE2E2, AARS1, PLP1, PSMD5, SFRP1, ZIC1, PHGDH, SLC7A3, LRRC55, SESN2, YARS1, EIF4EBP1, PMEL, CTH, PSPH, LHX5, REC8, SLC1A5, DPPA4, GARS1, FZD7, ATF4, CARS1, HERPUD1, XPOT, MTHFD1L, COL9A1, CEBPB, EIF2S2, ASS1, SLC3A2, IARS1, PCDHGC3, SLC7A1, RGS16, DOCK11, SMARCA1, WARS1, TNFRSF1A, TSC22D3, EFNB1, STAG2*
10	*FRZB, RCAN1, DYSF, MID1, MAPK8IP2, SMS, HSPA8, PFKL, PTTG1IP, LSS, TTC3*
